# Life Expectancy among Japanese of Different Smoking Status in Japan: NIPPON DATA80

**DOI:** 10.2188/jea.17.31

**Published:** 2007-04-10

**Authors:** Yoshitaka Murakami, Hirotsugu Ueshima, Tomonori Okamura, Takashi Kadowaki, Atsushi Hozawa, Yoshikuni Kita, Takehito Hayakawa, Akira Okayama, NIPPON DATA80 Research Group

**Affiliations:** 1 Department of Health Science, Shiga University of Medical Science.; 2 Department of Public Health, Shimane University.; 3 Department of Preventive Cardiology, National Cardiovascular Center.

**Keywords:** Life Expectancy, Smoking, Mortality, NIPPON DATA80

## Abstract

**BACKGROUND:**

The life expectancy is an important measure for describing health status among population. Several studies from the United States and Europe showed the harm of smoking by describing the life expectancies with different smoking status. No such study is examined in Japan, the country with the world s highest life expectancy irrespective of high smoking rate among men.

**METHODS:**

The abridged life table method was applied to calculate the life expectancies of men and women among different smoking status from age 40 until age 85. Age-specific mortality rates stratified by different smoking status were obtained from follow-up data from random sample in Japanese population (NIPPON DATA80).

**RESULTS:**

Proportion of current smokers was 62.9% in men and 8.8% in women at the baseline survey in 1980. The life expectancies of 40-year-old never smokers, ex-smokers and current smokers were 42.1, 40.4, and 38.6 years in men and 45.6, 45.9, and 43.4 years in women. The life expectancy of 40-year-old men who smoked less than one pack per day was 39.0 and was longer than that of those who smoked one or two packs (38.8) and more than two packs (38.1).

**CONCLUSION:**

Life expectancy decreased gradually as the grade of smoking increased in the Japanese population.

The harm of smoking has been known for many years^1^ and evidences from a cohort study shows that smoking is a prominent risk factor for total mortality,^2^^,^^3^ lung cancer,^4^^,^^5^ all cardiovascular disease,^6^^,^^7^ ischemic heart disease,^7^ and stroke^7^^,^^8^ in the Japanese population. Though the proportion of men's smoker in Japan has been quite high (52.8 % in year 1999) among the developed countries,^9^ the highest levels of life expectancy in men (78 years in 2002) have continued for a long period.^10^ This situation, so-called 'Japanese smoking paradox',^11^ is still matter of discussion. Though longevity in Japan is suffered from smoking, the difference of life expectancy between never smokers and current smokers is not yet examined in Japan. In order to activate public health measures for smoking cessation in Japan, it is necessary to show the impacts of smoking on health in an intelligible way. Life expectancy is a well-known comprehensive figure that represents the health status of a population. In the broad area of public health, life expectancy is used for comparing the health statuses of different groups.

The present study examined life expectancies among Japanese with different smoking statuses in Japan, which has world's highest life expectancy even though high proportion of smoking has been observed in men.

## METHODS

### Data Source

Age-specific mortality rates among Japanese with different smoking statuses were calculated from the data of the nationwide cohort study in Japan, called the NIPPON DATA80 (National Integrated Project for Prospective Observation of Non-communicable Disease And its Trends in the Aged). Baseline examination was conducted in year 1980 and the details of the study were described elsewhere.^71213^ Persons who were aged 30 and over in 1980 were enrolled by stratified random sampling throughout Japan, and a total of 10,546 participants (men: 4,639, women: 5,907) were followed until the end of 1999. The national vital statistics were used for ascertaining the vital status of participants. Of the 10,546 original participants, 921 participants were excluded from the present study because they were lost to follow-up (n=908) or because there was no smoking information in the baseline survey (n=13). The remaining 9,625 participants consist of 4,237 men and 5,388 women. Approval for this study was obtained from Institutional Review Board of Shiga University of Medical Science (no. 12-18, 2000). Study group members are listed in Reference 13.

The participants' profiles, including smoking status, were collected by the questionnaire in the baseline survey. The smoking status was classified into three categories: never smoking, ex-smoking, and current smoking. The amount of cigarette smoking per day was also classified into three categories: smoking less than one pack per day, smoking one or two packs per day, and smoking more than two packs per day.

### Statistical Analysis

Age-specific mortality rates were calculated with the person-year method.^14^ Age-specific mortality rates were calculated within five-year age categories. The age categories began at age 40 year and the highest age category was set at age 85 years and over. The age-specific mortality rates in men and women with different smoking statuses are shown in the appendix 1. Byer's method^15^ was used for calculating the 95 % confidence intervals.

**Table 1.** The basic characteristics of Japanese with different smoking status in the baseline survey in 1980, NIPPON DATA80, 1980-1999, Japan.

		Never smokers n (%)	Ex-smokers n (%)	Current smokers n (%)	Number of cigarette packs per day		

					<1 pack n (%)	1-2 packs n (%)	2+ packs n (%)
		Men (n=4,237)					
Total		777 (18.3)	794 (18.7)	2666 (62.9)	1635 (38.6)	880 (20.8)	151 (3.6)

Alcohol drinking	Non-drinkers	219 (28.2)	154 (19.4)	475 (17.8)	298 (18.2)	153 (17.4)	24 (15.9)
	Ex-drinkers	33 (4.2)	87 (11.0)	127 (4.8)	95 (5.8)	27 (3.1)	5 (3.3)
	Current	525 (67.6)	551 (69.4)	2061 (77.3)	1240 (75.8)	700 (79.5)	121 (80.1)
	Missing	0	2 (0.3)	3 (0.1)	2 (0.1)	0	1 (0.7)

Physical activity in daily living	Light	256 (36.3)	268 (33.8)	992 (37.2)	602 (36.8)	330 (37.5)	60 (39.7)
	Midium	282 (36.3)	260 (32.7)	974 (36.5)	607 (37.1)	327 (37.2)	40 (26.5)
	Heavy	209 (26.9)	264 (33.2)	696 (26.1)	424 (25.9)	222 (25.2)	50 (33.1)
	Missing	4 (0.5)	2 (0.3)	4 (0.2)	2 (0.1)	1 (0.1)	1 (0.7)

Residence	Urban	422 (54.3)	467 (58.8)	1447 (54.3)	857 (52.4)	501 (56.9)	89 (58.9)
	Rural	337 (43.4)	312 (39.3)	1138 (42.7)	723 (44.2)	356 (40.5)	59 (39.1)
	Others	18 (2.3)	12 (1.5)	74 (2.8)	51 (3.1)	20 (2.3)	3 (2.0)
	Missing	0	3 (0.4)	7 (0.3)	4 (0.2)	3 (0.3)	0

	Mean (SD)	Mean (SD)	Mean (SD)	Mean (SD)	Mean (SD)	Mean (SD)

Age at study entry (years)		50.6 (14.4)	53.3 (13.7)	49.0 (12.7)	51.2 (13.3)	45.9 (11.0)	44.3 (9.5)
Height (cm)		161.4 (7.2)	162.9 (30.5)	162.7 (17.5)	161.5 (6.6)	164.7 (28.9)	164.5 (5.8)
Weight (kg)		60.2 (9.3)	59.3 (9.3)	59.0 (9.1)	57.6 (8.9)	61.0 (9.0)	62.8 (9.1)



		Never smokers n (%)	Ex-smokers n (%)	Current smokers n (%)			

		Women (n=5,388)					
Total		4793 (89.0)	119 (2.2)	476 (8.8)			

Alcohol drinking	Non-drinkers	3916 (81.7)	61 (51.3)	258 (54.2)			
	Ex-drinkers	46 (1.0)	13 (10.9)	24 (5.0)			
	Current	825 (17.2)	45 (37.8)	194 (40.8)			
	Missing	6 (0.1)	0	0			

Physical activity in daily living	Light	871 (18.2)	27 (22.7)	62 (13.0)			
	Midium	1698 (35.4)	43 (36.1)	181 (38.0)			
	Heavy	2183 (45.5)	48 (40.3)	228 (47.9)			
	Missing	41 (0.9)	1 (0.8)	5 (1.1)			
Residence	Urban	2583 (53.9)	82 (68.9)	348 (73.1)			
	Rural	2086 (43.5)	37 (31.1)	113 (23.7)			
	Others	106 (2.2)	0	12 (2.5)			
	Missing	18 (0.4)	0	3 (0.6)			

	Mean (SD)	Mean (SD)	Mean (SD)			

Age at study entry (years)		50.2 (13.3)	54.4 (14.3)	51.2 (13.6)			
Height (cm)		149.9 (6.1)	150.3 (6.1)	150.3 (6.3)			
Weight (kg)		51.5 (8.3)	52.2 (8.3)	50.8 (9.1)			


SD: Standard deviation

The abridged life table method was used to calculate life expectancies. The fraction of the last age interval of life^16^^,^^17^ was used to construct an abridged life table. Those fractions were calculated from a complete life table for the year 1990 in Japan^18^ and those figures were shown in appendix 2. Each life expectancy was calculated from age 40 until age 85 by five-year interval. We also calculated 95 % confidence intervals of life expectancy in each age group. As the current smokers in women were so few that cannot examine the difference among the amount of cigarettes, life expectancies for participants with different amounts of cigarette smoking per day were only examined in men. The amount of cigarettes smoking was classified into three groups: less than one pack, one to two packs and more than two packs. To examine the differences from the general Japanese population, the overall life expectancies calculated from NIPPON DATA80 were compared with those from a complete life table for the year 1990 in Japan.18 All statistical analysis was performed using SAS® version 8.2 (SAS Institute, Cary, NC).

**Table 2.** Life expectancies of Japanese with different smoking status from NIPPON DATA80, 19 year follow-up, 1980-1999, Japan

Age group (year)	Complete life table (1990)	Smoking status				Cigarettes packs per day		
	
		Over all	Never smokers	Ex-smokers	Current smokers	<1 pack	1-2 packs	2+ packs
Men								
40	37.52	39.5 ( 39.0 - 40.1 )	42.1 ( 40.8 - 43.3 )	40.4 ( 39.3 - 41.6 )	38.6 ( 37.9 - 39.3 )	39.0 ( 38.2 - 39.8 )	38.8 ( 37.2 - 40.3 )	38.1 ( 34.5 - 41.6 )
45	32.85	34.8 ( 34.3 - 35.3 )	37.5 ( 36.4 - 38.6 )	35.4 ( 34.3 - 36.6 )	33.9 ( 33.2 - 34.5 )	34.2 ( 33.4 - 35.0 )	34.1 ( 32.6 - 35.7 )	33.1 ( 29.5 - 36.6 )
50	28.33	30.2 ( 29.8 - 30.7 )	32.6 ( 31.5 - 33.7 )	30.9 ( 29.8 - 31.9 )	29.4 ( 28.8 - 30.1 )	29.7 ( 29.0 - 30.4 )	29.8 ( 28.2 - 31.3 )	28.7 ( 25.2 - 32.3 )
55	23.99	25.6 ( 25.1 - 26.1 )	27.8 ( 26.7 - 28.9 )	26.5 ( 25.5 - 27.5 )	24.8 ( 24.2 - 25.4 )	25.0 ( 24.3 - 25.7 )	25.2 ( 23.6 - 26.7 )	24.3 ( 20.7 - 27.8 )
60	19.95	21.5 ( 21.0 - 21.9 )	23.8 ( 22.8 - 24.8 )	22.1 ( 21.2 - 23.0 )	20.7 ( 20.1 - 21.3 )	20.8 ( 20.1 - 21.5 )	21.1 ( 19.6 - 22.7 )	20.4 ( 16.8 - 23.9 )
65	16.16	17.4 ( 16.9 - 17.8 )	19.3 ( 18.3 - 20.2 )	17.7 ( 16.8 - 18.5 )	16.8 ( 16.2 - 17.3 )	16.9 ( 16.2 - 17.5 )	17.4 ( 15.8 - 19.0 )	16.0 ( 12.5 - 19.6 )
70	12.60	13.6 ( 13.2 - 14.0 )	14.9 ( 14.0 - 15.8 )	13.8 ( 13.0 - 14.6 )	13.1 ( 12.6 - 13.7 )	13.0 ( 12.5 - 13.6 )	14.2 ( 12.6 - 15.8 )	13.2 ( 9.5 - 16.9 )
75	9.44	10.3 ( 10.0 - 10.7 )	11.3 ( 10.6 - 12.1 )	10.2 ( 9.5 - 11.0 )	10.1 ( 9.6 - 10.6 )	9.8 ( 9.3 - 10.4 )	12.0 ( 10.3 - 13.7 )	9.6 ( 5.8 - 13.4 )
80	6.82	7.5 ( 7.2 - 7.8 )	7.7 ( 7.1 - 8.3 )	7.6 ( 6.9 - 8.2 )	7.4 ( 7.0 - 7.9 )	7.1 ( 6.6 - 7.5 )	10.4 ( 8.8 - 12.1 )	7.3 ( 3.0 - 11.6 )
85	4.82	5.5 ( 4.8 - 6.2 )	5.2 ( 4.0 - 6.4 )	5.7 ( 4.2 - 7.3 )	5.6 ( 4.5 - 6.7 )	5.1 ( 4.0 - 6.1 )	9.2 ( 3.8 - 14.6 )	6.5 ( -- - )

Women								
40	42.90	45.4 ( 44.9 - 45.8 )	45.6 ( 45.1 - 46.1 )	45.9 ( 43.1 - 48.6 )	43.4 ( 41.7 - 45.2 )			
45	38.12	40.6 ( 40.1 - 41.0 )	40.8 ( 40.3 - 41.3 )	40.9 ( 38.1 - 43.6 )	38.7 ( 37.0 - 40.4 )			
50	33.41	35.8 ( 35.4 - 36.3 )	36.0 ( 35.6 - 36.5 )	35.9 ( 33.1 - 38.6 )	33.9 ( 32.2 - 35.5 )			
55	28.80	31.1 ( 30.7 - 31.6 )	31.3 ( 30.9 - 31.8 )	30.9 ( 28.1 - 33.6 )	29.3 ( 27.7 - 30.9 )			
60	24.29	26.6 ( 26.2 - 27.0 )	26.8 ( 26.3 - 27.2 )	25.9 ( 23.1 - 28.6 )	25.2 ( 23.7 - 26.7 )			
65	19.92	22.1 ( 21.7 - 22.5 )	22.3 ( 21.9 - 22.8 )	21.3 ( 18.7 - 24.0 )	20.5 ( 19.1 - 22.0 )			
70	15.76	17.9 ( 17.5 - 18.3 )	18.0 ( 17.6 - 18.4 )	17.5 ( 15.0 - 20.0 )	16.9 ( 15.6 - 18.3 )			
75	11.95	14.0 ( 13.6 - 14.3 )	14.0 ( 13.6 - 14.4 )	13.8 ( 11.4 - 16.2 )	13.9 ( 12.6 - 15.1 )			
80	8.60	10.6 ( 10.3 - 10.9 )	10.5 ( 10.1 - 10.8 )	11.9 ( 9.9 - 13.9 )	11.3 ( 10.3 - 12.3 )			
85	5.90	8.0 ( 7.0 - 8.9 )	7.9 ( 6.9 - 8.8 )	9.7 ( 2.9 - 16.6 )	8.5 ( 5.2 - 11.8 )			

Over all: Life expectancies of all participants were calculated by using age-specific mortality rates regardless of smoking status

(Data of never smokers, ex-smokers and current smokers were all combined).

## RESULTS

Table 1 shows the basic characteristics of participants with different smoking statuses in the baseline survey. The proportion of current smokers in the baseline survey was 62.9 % in men and 8.8 % in women. In men, the proportion of non-drinkers among never smokers (28.2 %) was slightly larger than that among current smokers (17.8 %) and among ex-smokers (19.4 %), but no difference was found in other characteristics (physical activity of daily living, residence, age at entry, height and weight). In women, the proportion of non-drinkers among never smokers (81.7 %) was greater than that among current smokers (54.2 %) and among ex-smokers (51.3 %), and the proportion of current smokers living in urban areas (73.1 %) was larger than that of never smokers (53.9 %). No apparent difference was found in physical activity of daily living, age at entry, height and weight.

Table 2 shows the life expectancies among participants with different smoking statuses from age 40 year until age 85 year and over. Life expectancies in 40-year-old men and women were 42.1 years and 45.6 years in never smokers, 40.4 years and 45.9 years in ex-smokers, and 38.6 years and 43.4 years in current smokers, respectively. The life expectancies of 40-year-old never smokers were longer than those of current smokers in both men and women. This order of life expectancy with respect to smoking status was seen in most age groups with a few exceptions.

The life expectancy of men who smoked less than one pack per day was longer than that of men who smoked more than two packs per day. Though there was some fluctuation, the life expectancies of people who smoked one or two packs per day were slight longer than other groups from age 50.

The overall life expectancy of 40-year-old participants, regardless of smoking status, was 39.5 years in men and 45.4 years in women. Those figures were larger than the life expectancies from the complete life table in Japan in 1990, which are 37.52 years in men and 42.90 years in women.^18^ This difference was seen consistently in all age groups in both men and women.

## DISCUSSION

The current study presented the life expectancies among different smoking statuses determined from the data in the nationwide cohort study in Japanese population. The results showed that the life expectancy of current smokers was two or three years shorter than that of ex-smokers and of never smokers in both men and women in most age categories. Among men's current smokers, the life expectancy of those who smoked less than one pack per day was longer than that of smokers who smoked more than two packs per day.

Our finding is applicable to general Japanese population. The cohort of NIPPON DATA80 was selected initially by stratified random sampling. This participant selection ensured that our result also apply to the general Japanese population without difficulty. Comparing our results to the complete life table for the same period in Japan, the life expectancies that we determined were longer than those from the complete life table. Participants lost to follow-up in the cohort were excluded from our analysis. In the baseline survey, people with health problems, such as inpatients or people with recuperation, could not participate in the survey. That may have caused age-specific mortality rates to move downwards and affect the difference of life expectancies. The stable population of final age interval (age 85 and over) is calculated number of survivors of 85 years old divided by death rate of 85 years and over. Though this is usual way to deal the final age interval in the calculation of a life expectancy, it may overestimate the life expectancy.^17^ This overestimation also influences the difference between our results and those from complete life table. Though the absolute life expectancies were different, relative orders of life expectancies with respect to smoking status might be the same.

Though there were some exceptions in specific age categories, there was a consistent order of life expectancy with respect to the smoking status. There was a two or three year difference between never smokers and current smokers, and a two year difference between never smokers and ex-smokers in both men and women. Among three categories on the amount of cigarette packs, decrease of life expectancies of 40 year-old was observed as the amount of cigarette increase. This tendency came obscure when we observed life expectancy of the older age group. Small sample was only available in older age group and that reflected the wide 95 % confidence interval of life expectancies on older age group. We should be caution when we discussed about the life expectancy in older age group.

Several studies found a harm of smoking by using life expectancy and other related measures. In the United States, the life expectancy of 40-year-old non-smokers, former and current smokers are 38.7, 37.5, and 31.8 years in men, and 46.1, 42.9, and 39.3 years in women.^19^ The multi-state life table data of the Framingham Heart Study reveals that the differences of life expectancies between 50-years-old always-smokers and never-smokers are 8.66 year in men and 7.59 years in women.^20^ In Australia, the difference of life expectancies of 15 year-old men between non-smokers and current smokers was 5.6 years in mid 1980-1990.^21^ In Denmark, the cohort life table technique was applied to find that the differences in median survivals between non-smokers and heavy smokers were 9.2 years in men and 9.4 years in women.^22^ These consistent differences of life expectancy between never smokers and current smokers were found in all studies.

Our findings of the differences in life expectancies between never smokers and current smokers are smaller than those of other articles.^19^^-^^22^ In addition to the dilution problem we mentioned bellow, these smaller differences might be a reflection of the circumstances in Japan in 1980. In men, the smoking habit was so widespread in 1980 and some never smokers probably had other health problems. Also, smoking by women was not popular at that time, especially for housewives. This circumstance is quite different from other developed countries in those days, and this could account for the smaller differences in life expectancies with respect to smoking status in Japan. Comparison of life expectancies between current smokers with never smokers in Japan may be considered as the contrast between men's current smokers and those never smokers mixed with unhealthy people, and also the contract between women's current smokers with vitality and women in common. This might due to the relatively small difference in life expectancies compared with those of other developed countries. In the circumstance of Japan around 1980, separation of smoking areas was insufficient and passive smoking in the household was speeded. That may also causes the relatively small difference in life expectancies among never-smokers and current smokers.

Possible misclassification of smoking categories might influence our results. Classification of smoking status was only made with the smoking information in the baseline survey, and we assumed that the smoking status of individuals did not change during the follow-up period. This assumption would be violated if smokers quit smoking or never smokers started smoking. Some people might make false statements in the survey. It is not certain how much change of smoking habit would occur during a 19-year period and how many people did not answer the true condition of smoking. Even though, the influence of this misclassification might dilute the difference of life expectancies among groups and that might make our results conservative.

We recognize that all the differences in life expectancies are not caused by smoking, and other factors would also affect the mortality rate. There was no distinct difference in baseline characteristics between the three different smoking statuses in men. The differences in life expectancy might come mainly from smoking; whereas in women, some characteristics such as drinking status and residence were different between never smokers and smokers (former and current) in our study. As mentioned above, the smoking experience among Japanese women was not so common in 1980 and such experience was highly associated with life style and health behavior. In addition to smoking, other factors probably influence on life expectancy in women.

In conclusion, life expectancies of participants with different smoking statuses were examined using data from a nationwide cohort study in Japan. A gradual decrease in life expectancy was observed as the grade of smoking increased in Japan. A two or three years gain in life expectancy could still be possible if the smoke free society were established in Japan, the country with the world's highest life expectancy.

**Appendix 1.** Age specific mortality rates of Japanese with different smoking statuses from NIPPON DATA80, 19 year follow-up, 1980-1999, Japan.

Age-specific mortality rates (per 1,000) among never smokers, ex-smokers and current smokers, 1980-1999, Japan									

	Never smokers			Ex-smokers			Current smokers		
		
Age (year)	No. of deaths	Person-years	Molality rate (/1000) (95% confidence interval)	No. of deaths	Person-years	Molality rate (/1000) (95% confidence interval)	No. of deaths	Person-years	Motalityrate(/1000) (95% confidence interval)

Men									

30-34	0	290.0	0.0 ( 0.0 - 8.5 )	0	250.0	0.0 ( 0.0 - 9.9 )	2	1207.8	1.7 ( 0.3 - 5.3 )
35-39	0	754.0	0.0 ( 0.0 - 3.3 )	0	553.3	0.0 ( 0.0 - 4.5 )	2	3005.8	0.7 ( 0.1 - 2.1 )
40-44	3	1309.8	2.3 ( 0.6 - 6.1 )	0	1017.0	0.0 ( 0.0 - 2.4 )	6	4671.9	1.3 ( 0.5 - 2.6 )
45-49	1	1896.5	0.5 ( 0.0 - 2.5 )	4	1448.2	2.8 ( 0.9 - 6.6 )	23	6173.6	3.7 ( 2.4 - 5.5 )
50-54	3	1995.9	1.5 ( 0.4 - 4.0 )	7	1649.7	4.2 ( 1.9 - 8.3 )	17	6469.7	2.6 ( 1.6 - 4.1 )
55-59	14	1795.9	7.8 ( 4.5 - 12.7 )	9	1733.3	5.2 ( 2.6 - 9.5 )	48	6280.7	7.6 ( 5.7 - 10.0 )
60-64	6	1441.7	4.2 ( 1.7 - 8.6 )	9	1614.9	5.6 ( 2.8 - 10.2 )	66	5560.7	11.9 ( 9.3 - 15.0 )
65-69	8	1153.1	6.9 ( 3.3 - 13.1 )	23	1535.4	15.0 ( 9.8 - 22.1 )	86	4682.8	18.4 ( 14.8 - 22.6 )
70-74	21	935.2	22.5 ( 14.3 - 33.7 )	31	1301.1	23.8 ( 16.5 - 33.4 )	115	3392.8	33.9 ( 28.1 - 40.5 )
75-79	22	762.4	28.9 ( 18.6 - 42.9 )	53	1014.5	52.2 ( 39.6 - 67.8 )	115	2107.9	54.6 ( 45.3 - 65.2 )
80-84	43	544.9	78.9 ( 57.9 -105.2 )	59	614.8	96.0 ( 73.8 -122.9 )	107	1112.1	96.2 ( 79.3 -115.8 )
85+	56	292.1	191.7(146.3 -247.0 )	45	258.4	174.2 (128.7 -230.8 )	85	474.7	179.1 (144.0 -220.3 )

Women									

30-34	0	1939.3	0.0 ( 0.0 - 1.3 )	0	39.0	0.0 ( 0.0 - 63.2 )	0	184.9	0.0 ( 0.0 - 13.3 )
35-39	1	4880.7	0.2 ( 0.0 - 1.0 )	0	113.0	0.0 ( 0.0 - 21.8 )	0	474.6	0.0 ( 0.0 - 5.2 )
40-44	8	7919.5	1.0 ( 0.5 - 1.9 )	0	149.4	0.0 ( 0.0 - 16.5 )	1	744.4	1.3 ( 0.1 - 6.3 )
45-49	13	10723.5	1.2 ( 0.7 - 2.0 )	0	186.0	0.0 ( 0.0 - 13.2 )	1	1000.0	1.0 ( 0.1 - 4.7 )
50-54	20	11396.5	1.8 ( 1.1 - 2.7 )	0	205.0	0.0 ( 0.0 - 12.0 )	3	1021.1	2.9 ( 0.8 - 7.8 )
55-59	34	11143.2	3.1 ( 2.2 - 4.2 )	0	188.0	0.0 ( 0.0 - 13.1 )	6	954.7	6.3 ( 2.6 - 13.0 )
60-64	49	10339.2	4.7 ( 3.5 - 6.2 )	1	244.6	4.1 ( 0.4 - 19.1 )	3	961.2	3.1 ( 0.9 - 8.3 )
65-69	61	8963.0	6.8 ( 5.3 - 8.7 )	3	249.9	12.0 ( 3.3 - 32.0 )	14	933.3	15.0 ( 8.6 - 24.5 )
70-74	88	7101.9	12.4 ( 10.0 - 15.2 )	4	236.9	16.9 ( 5.6 - 40.1 )	19	739.7	25.7 ( 16.0 - 39.3 )
75-79	119	4956.7	24.0 ( 20.0 - 28.6 )	9	185.3	48.6 ( 24.0 - 88.7 )	21	546.1	38.5 ( 24.5 - 57.7 )
80-84	163	3087.7	52.8 ( 45.1 - 61.4 )	6	114.2	52.5 ( 21.8 -108.3 )	15	335.1	44.8 ( 26.2 - 72.0 )
85+	225	1766.5	127.4(111.5 -144.8 )	7	68.2	102.6 ( 45.8 -201.5 )	22	186.6	117.9 ( 76.0 -175.3 )


Age-specific mortality rates (per 1,000) of current smokers by number of cigarettes smoked per day (Men), 1980-1999, Japan									

	<1 pack			1-2 pack			2+ pack		
		
Age (year)	No. of deaths	Person-years	Molality rate (/1000) (95% confidence interval)	No. of deaths	Person-years	Molality rate (/1000) (95% confidence interval)	No. of deaths	Person-years	Motalityrate(/1000) (95% confidence interval)


Men									

30-34	1	669.4	1.5 ( 0.1 - 7.0 )	1	476.4	2.1 ( 0.2- 9.8)	0	62.0	0.0 ( 0.0 - 39.7 )
35-39	0	1638.2	0.0 ( 0.0 - 1.5 )	1	1144.6	0.9 ( 0.1 - 4.1 )	1	223.1	4.5 ( 0.4 - 20.9 )
40-44	2	2439.7	0.8 ( 0.2 - 2.6 )	4	1876.2	2.1 ( 0.7 - 5.1 )	0	356.0	0.0 ( 0.0 - 6.9 )
45-49	11	3237.1	3.4 ( 1.8- 5.9)	10	2469.2	4.0 ( 2.1 - 7.2 )	2	467.3	4.3 ( 0.9 - 13.7 )
50-54	8	3442.8	2.3 ( 1.1 - 4.4 )	7	2537.8	2.8 ( 1.2 - 5.4 )	2	489.2	4.1 ( 0.8 - 13.1 )
55-59	24	3546.6	6.8 ( 4.4 - 9.9 )	20	2345.1	8.5 ( 5.4 - 12.9 )	4	389.0	10.3 ( 3.4 - 24.4 )
60-64	40	3452.5	11.6 ( 8.4 - 15.6 )	24	1818.6	13.2 ( 8.7 - 19.3 )	2	289.6	6.9 ( 1.4 - 22.1 )
65-69	51	3208.4	15.9 ( 12.0 - 20.7 )	29	1276.6	22.7 ( 15.5 - 32.2 )	6	197.8	30.3 ( 12.6 - 62.5 )
70-74	80	2527.3	31.7 ( 25.3 - 39.2 )	32	742.8	43.1 ( 30.0 - 60.0 )	3	122.7	24.4 ( 6.8 - 65.2 )
75-79	89	1686.9	52.8 ( 42.6 - 64.6 )	22	358.4	61.4 ( 39.6 - 91.3 )	4	62.6	63.9 ( 21.4 -151.9 )
80-84	91	913.1	99.7 ( 80.7 -121.8 )	14	182.8	76.6 ( 43.9 -125.1 )	2	16.2	123.3 ( 24.6 -395.2 )
85+	75	380.8	197.0 (156.1 -245.5 )	10	91.9	108.8 ( 55.8 -193.0 )	0	2.0	0.0 ( 0.0 -1232.0)

**Appendix 2.** The fraction of the last age interval of life from Vital statistics in 1990, Japan.

Age (year)	Men	Women
40-44	0.537	0.533
45-49	0.537	0.539
50-54	0.542	0.526
55-59	0.534	0.534
60-64	0.525	0.532
65-69	0.528	0.541
70-74	0.531	0.542
75-79	0.523	0.541
80-84	0.501	0.528
85-89	0.470	0.499
